# Application of a Smart Orthosis in the Treatment of Idiopathic Scoliosis—A Pilot Case Study

**DOI:** 10.3390/s26103169

**Published:** 2026-05-17

**Authors:** Patrycja Tymińska-Wójcik, Katarzyna Zaborowska-Sapeta, Tomasz Giżewski

**Affiliations:** 1 Department of Electrical Devices and High Voltage Technology, Lublin University of Technology, Nadbystrzycka 38d, 20-618 Lublin, Poland; 2 Department of Rehabilitation and Orthopedics, School of Medicine, University of Warmia and Mazury in Olsztyn, Michała Oczapowskiego 2, 10-719 Olsztyn, Poland; katarzyna.zaborowska@uwm.edu.pl; 3 Regional Specialized Children’s Hospital in Olsztyn, Żołnierska 18a, 10-561 Olsztyn, Poland; 4 Department of Electrical Engineering and Superconducting Technologies, Lublin University of Technology, Nadbystrzycka 38d, 20-618 Lublin, Poland; t.gizewski@pollub.pl

**Keywords:** brace, scoliosis, force sensors, wearable devices

## Abstract

The increasing demand for personalized conservative treatment of idiopathic scoliosis (IS) highlights the need for objective and continuous monitoring of corrective forces during brace therapy. This study aims to evaluate the feasibility and clinical relevance of a smart orthopedic brace equipped with integrated force sensors for long-term biomechanical assessment. Three female patients with different types of idiopathic scoliosis were treated using a custom-designed thoracolumbosacral orthosis incorporating four flexible pressure sensors, enabling real-time and long-term recording of corrective forces at key anatomical locations. Sensor data were analyzed in relation to brace-wearing adherence, patient activity, and radiological outcomes assessed using Cobb angle measurements. The results demonstrated substantial variability in force distribution and wearing patterns among patients, which was associated with differences in treatment effectiveness. Higher and more stable corrective forces near curve apices were generally accompanied by improved radiological outcomes, whereas irregular brace use and uneven pressure distribution limited therapeutic effects. Long-term monitoring enabled identification of insufficient correction zones and adherence issues. In conclusion, the proposed sensor-based orthotic system provides clinically relevant information on force distribution and brace use, supporting individualized therapy optimization. These findings indicate that smart braces can enhance clinical decision-making and contribute to more effective and personalized scoliosis management.

## 1. Introduction

Idiopathic scoliosis (IS) is a three-dimensional spinal deformity that affects a significant proportion of the pediatric and adolescent population. Recent data analyses indicate a prevalence of 3.1% in this age group [1]. Its treatment often requires prolonged use of an orthopedic brace, where precise adjustment of corrective forces and continuous monitoring of therapeutic effectiveness are of critical importance. In this context, the development of smart orthoses equipped with data acquisition systems has become a key research direction. The etiopathogenesis of IS remains complex and multifactorial. Recent genetic and molecular studies have identified the involvement of numerous genes related to connective tissue development, mechanotransduction, and growth regulation, as well as their interactions with environmental factors [2]. These findings suggest that scoliosis is not a uniform disease but rather a spectrum of disorders with diverse pathogenic mechanisms, which further underscores the need for individualized therapeutic approaches.

In this study, we present a smart orthopedic brace designed to record the distribution of corrective forces acting on the patient’s body during everyday use. The information obtained in this way enables not only an objective assessment of the therapy’s progress but also the analysis of individual physiological responses depending on the degree of deformity and the patient’s activity level. The approach, combining personalized therapy and real-world data analysis, provides a basis for future AI-based methods. Our study presents a case analysis in which a smart orthosis with an integrated data acquisition system was used to enable long-term monitoring of therapy in clinical conditions.

The Society on Scoliosis Orthopaedic and Rehabilitation Treatment (SOSORT) guidelines, published in 2016, specify indications for treating IS in growing patients with orthopedic braces [3]. The recommendations highlight the importance of using the most validated methods and conducting individualized assessments of treatment effectiveness based on objective data. Most proposed technological solutions focus on adherence monitoring [4,5] justified by the strong link between brace wear duration and therapy success. While the length of brace wear is a well-documented factor affecting curvature correction, it is not the only one. The distribution and amount of forces are also critical. Poor distribution of corrective forces, even with full orthosis wear, will not lead to an effective therapeutic outcome. So far, pressure sensors based on piezoresistive polymers, textile matrices, or dielectric elastomers have been developed to monitor the magnitude and distribution of forces exerted by the brace on the torso [6,7,8].

This article presents selected results from the analysis of corrective forces acting in a smart orthopedic brace used for patients with IS. The study focuses on evaluating the distribution of corrective forces and monitoring therapy adherence using integrated sensors. By combining radiological assessment with real-world data from the orthoses, this work aims to illustrate how personalized monitoring can support clinical decision-making and enhance understanding of treatment effectiveness in IS. This work is interdisciplinary in the field of medical research and electrical engineering, electronics, and computer science. In further stages of the project, the collected data are intended to support the development of artificial intelligence–based methods. In this article, we focus solely on the clinical results obtained, which provide a knowledge base for future AI applications.

The primary aim of this study was not to evaluate the long-term clinical effectiveness of brace therapy, which is already established in standard practice. Instead, the study represents a pilot technical investigation focused on testing the functionality of the integrated force-sensor system and analyzing the distribution of corrective forces and patient adherence during real-world brace use. Therefore, the aim of this pilot study was to investigate whether a thoracolumbosacral orthosis equipped with integrated force sensors can provide objective information on corrective force distribution and brace adherence during everyday use in patients with idiopathic scoliosis.

## 2. Materials and Methods

### 2.1. Participants

Three female patients with IS were prospectively included in this pilot case series. Inclusion criteria were: diagnosis of IS, indication for brace treatment according to the 2016 guidelines of the SOSORT, Risser stage (European version) 0–3, and Cobb angle between 20° and 35°. Exclusion criteria included previous spinal surgery or neuromuscular disorders. The number of patients (*n* = 3) reflects the preliminary and feasibility character of this study, aimed at evaluating the technical and clinical applicability of the smart brace system in real-life conditions. Due to inclusion criteria and the limited availability of patients meeting the requirements, three female patients were included in the study. This corresponds to the pilot nature of the study and allows assessment of the feasibility of the measurement system.

All patients received treatment according to SOSORT standards. They were prescribed scoliosis-specific exercises to be performed daily under physiotherapist guidance. Psychological support was not provided as none of the patients required it. Brace wear was prescribed for 23 h per day.

### 2.2. Brace and Sensor System

The case study includes three clinical patients who were fitted with orthosis equipped with four sensors recording pressure distribution in key regions of the spine and thorax. The brace used in this study was a rigid monocoque TLSO, manufactured by Technika Ortopedyczna Antoni Wylenzek (Wilkowice, Poland), with anterior opening, designed for three-dimensional correction based on the Chêneau concept, classified according to SOSORT brace classification parameters [9]. Each brace was individually molded to the patient using a traditional plaster cast method to obtain an exact body impression.

Four flexible force sensors were embedded at strategic locations on the inner surface of each brace to monitor corrective pressure distribution. The sensors had an accuracy of 1 N and recorded data at 1 Hz. Sensor placement was initially standardized but adapted for individual patient anatomy. Optimal sensor positioning was crucial to ensure precise measurements and effective action of the derotating pads supporting spinal curvature correction. The locations were selected based on biomechanical analysis and individual patient characteristics, with guidance from the attending physician, who relied on their knowledge and clinical experience, enabling therapy personalization. In all three patients, sensors were placed in comparable regions, taking into account individual anatomical differences. One sensor was positioned on the anterior thoracic wall on the left side to monitor forces acting on the upper torso, a region critical for stabilizing the thoracic spine. Another sensor was placed below the right scapula, close to the spine, enabling direct recording of forces near the apex of the curvature and allowing control of vertebral rotation. A third sensor was located laterally, also below the right scapula, to capture variations in pressure within the lateral deformity region. The fourth sensor was positioned below the left scapula, opposite the main curvature, providing complementary information about force distribution across both sides of the torso. The measurement system allowed real-time data acquisition during daily brace wear and aggregation of weekly and monthly summaries. Patients were instructed to wear the brace for at least 23 h per day, and adherence was indirectly estimated based on sensor-recorded forces.

The analysis was based on imaging data, including X-rays taken before and during therapy, to assess the degree of spinal deformity and treatment effectiveness. Sensor-recorded force data were complemented by charts showing pressure distribution, enabling detailed tracking of curvature correction dynamics over time. Mean, maximum, and minimum forces were analyzed with respect to sensor location and phase of therapy. The analysis also accounted for variations in patient activity and potential errors due to incorrect brace fastening. All measurements were evaluated both globally, using daily and monthly summaries, and locally, for individual sensors. This approach provided a detailed assessment of the brace’s effectiveness across different regions of the patient’s body and offered insight into how corrective forces vary according to patient activity and treatment progress.

#### Force Measurement System

The force sensor technology used in this study was an extension of the solutions described in [10], where graphene-based sensors were applied for continuous force measurement in orthopedic braces. In the present study, a similar concept was expanded to include long-term clinical monitoring of three patients with different types of IS. Each patient wore the smart brace with integrated force sensors for a minimum period of three months. The collected data were analyzed in terms of temporal pressure distribution, adherence to wearing instructions, and corrective changes measured by the Cobb angle.

**Figure 1:**
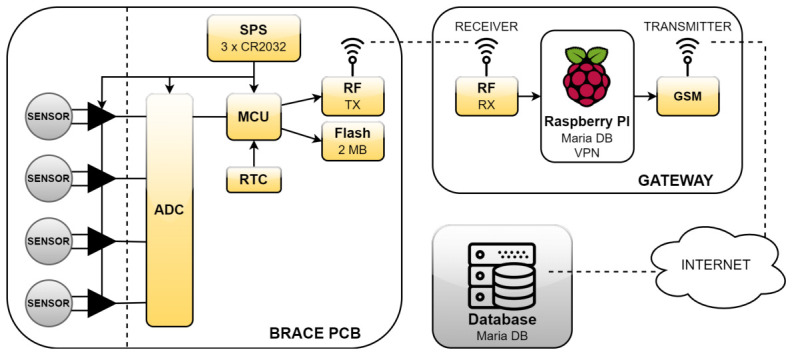
Diagram of the measurement system.

**Figure 2:**
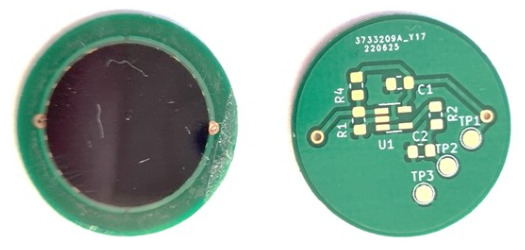
Graphene-based force sensor embedded within the brace measurement system.

The initial tests revealed errors in the measurement system, both in the software and the electronic components (the system described in [10]). A new measurement system was developed with wireless data transmission and a design of thin-film pressure sensors (Figure 1). Problems from the first system, such as battery discharge requiring patient intervention and intermittent GSM connection causing loss of measurement data, were eliminated in the new system. Sensors were designed and fabricated with a measurement surface integrated with the electronic circuit using a Wheatstone bridge (Figure 2). This allowed a significant reduction in sensor dimensions, saving space in the orthopedic brace and improving user comfort. Efforts focused on optimizing the system to be maintenance-free for the patient: no recharging was required, unlike in the first stage of measurements. Due to the custom-designed low-power PCB, the system could operate for over a month on a single set of batteries, which were replaced during each follow-up visit.

The new system (Figure 1) consisted of two parts: the first installed in the brace, a measurement unit with an analog-to-digital converter and thin-film pressure sensors and the second, a gateway consisting of a receiver and transmitter. Measurements were transmitted wirelessly to this device, which then used a Raspberry Pi to send the data via the GSM network to the database. This solution eliminated the need to recharge the device in the brace or manually enter data into the database. It also allowed regular collection of new measurements—daily, not only during control visits.

The system operated as follows: the real-time clock awakened the processor from sleep every second; the processor retrieved the current date and time from the clock, performed the measurements, and then transmitted them to the gateway device. The gateway then sent the data to the database. Raw data stored in the database were processed using LabVIEW and subsequently saved back to the database. The processed data were visualized using Lazarus in the form of box plots.

Each sensor was fabricated manually and individually calibrated. Calibration used a mechanical setup capable of applying forces from 0 to 450 N. Force–voltage characteristics were recorded for each sensor, and calibration curves were used to convert analog voltage readings from the microcontroller into force values. Sensors were thin-film graphene-based devices, consisting of a graphene layer on one film and a silver electrode layer on a second film, bonded together with adhesive tape. Calibration and validation proceeded in two stages. First, preliminary classification was performed under DC excitation to reflect battery-powered operation. Second, high-precision calibration was performed on the best sensors using AC excitation with frequencies from 40 Hz to 2 MHz [10].

Compared to the system described in [10], this setup extended the duration of measurements and included a larger number of patients. The measurement system was redesigned into two separate modules, which improved energy efficiency and eliminated the need for battery recharging. Sensor design was slightly modified to optimize integration with the new electronics. At this stage, measurements were collected without an active feedback system, as the data were intended for building a decision-making framework based on machine learning and artificial intelligence. This approach allowed the accumulation of high-quality, longitudinal data necessary for developing predictive models and evaluating brace performance across multiple patients over extended periods. Key improvements over the previous prototype are summarized in Table 1, highlighting enhancements in monitoring duration, data volume, power management, system architecture, data transmission, clinical validation, and application goals. These enhancements demonstrate that, while the sensor core builds on prior work, the current system represents a substantial advance in usability, reliability, and clinical applicability.

### 2.3. Radiographic Assessment

Spinal deformity was evaluated using standing posteroanterior and lateral X-rays taken before brace application and at follow-up intervals during therapy. Cobb angles and vertebral rotation at curve apices were measured, and skeletal maturity was assessed with the Risser scale. Apical vertebral rotation was estimated on standard radiographs during routine clinical evaluation using a qualitative Cobb-based approach, based on the position of the spinous process relative to the vertebral body. In this method, the vertebral body is conceptually divided into four equal regions, allowing classification of rotation into graded levels. This qualitative assessment is commonly applied in clinical practice as a rapid estimation of the rotational component of the deformity [11,12]. Imaging provided objective reference points to correlate sensor-recorded corrective forces with clinical outcomes.

### 2.4. Brace Adherence Assessment

Brace adherence was estimated indirectly based on sensor-recorded corrective forces. Periods during which at least one sensor recorded forces above the predefined threshold were interpreted as effective brace wear. A force threshold of 5 N was used to define effective brace wear: any period during which the measured force at least one sensor exceeded 5 N was considered as active wear. Interruption periods longer than 30 min, during which no sensor recorded force above the threshold, were classified as non-wear. Sensor malfunction was distinguished from true non-adherence by verifying data consistency across all sensors and system diagnostic signals. Adherence was calculated as the percentage of the recommended wear time (23 h/day) during which the brace was actively worn:

$$\mathrm{Adherence}\;\left(\%\right)=\frac{\mathrm{Total}\;\mathrm{time}\;\mathrm{with}\;F\geq F_{\mathrm{thresh}}}{\mathrm{Total}\;\mathrm{prescribed}\;\mathrm{brace}\text{-}\mathrm{wearing}\;\mathrm{time}}\times 100$$

### 2.5. Case Reports

Each patient case is summarized below, highlighting spinal deformity, Risser stage, Cobb angles, vertebral rotation, brace protocol, and sensor placement. Figures show radiographs and brace sensor configuration.

#### 2.5.1. Case 1: Single-Curve Scoliosis

**Figure 3:**
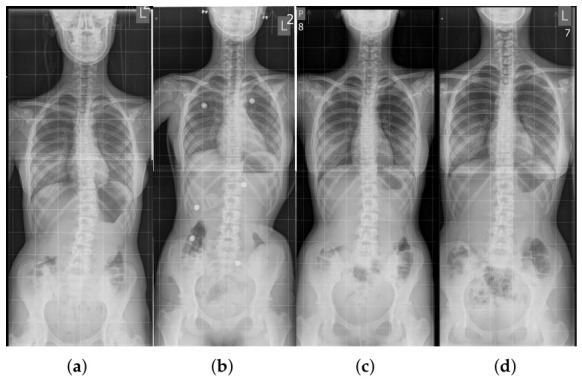
X-ray image of the spine of the first patient: (**a**) At the start of treatment. (**b**) While wearing the orthopedic brace. (**c**) During a follow-up visit in the course of therapy. (**d**) Without the brace, correction maintained.

A 13-year-old female patient was diagnosed with IS based on X-ray imaging. The spinal curvature, determined using the Cobb method, measured 25° in the Th11-L4 segment (Figure 3a,b). In-brace radiograph (Figure 3b) showed a reduction in the Cobb angle to 10°, indicating substantial immediate correction. Bone maturity, assessed using the Risser scale at level 3, indicated an intermediate stage of ossification and a risk of further deformity progression. The maximum vertebral rotation, assessed at the apical vertebra using a qualitative Cobb-based method (based on the position of the spinous process relative to the vertebral body), reached grade 4 [11,12], indicating a pronounced rotational component of the deformity.

Due to these factors, treatment with an orthopedic brace was initiated. At a follow-up visit after six months of therapy (Figure 3c), a significant improvement was observed: the Cobb angle decreased to 9°, indicating high treatment efficacy and proper adjustment of corrective forces. Radiological assessment was performed after a 24-h discontinuation of brace use, which did not reveal a persistent correction of the curvature.

At the end of therapy, complete correction of the Cobb angle was documented on X-ray. Vertebral rotation decreased to grade 2, indicating structural improvement of the spine. Importantly, the correction was maintained even after brace removal (Figure 3d), confirming the effectiveness of the treatment.

**Figure 4:**
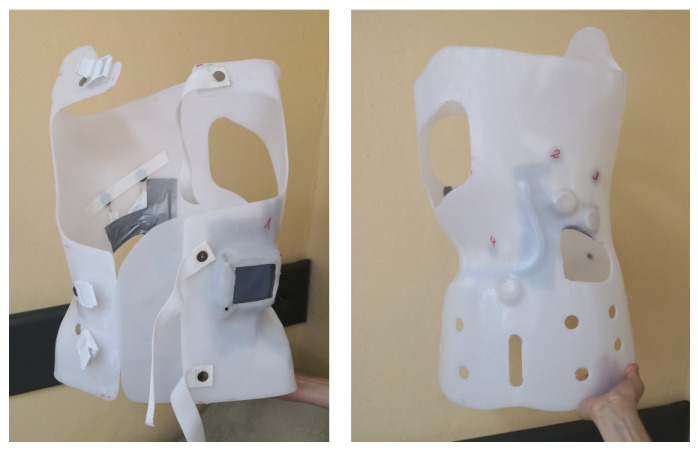
Temporary brace of the first patient with sensor placement indicated.

**Figure 5:**
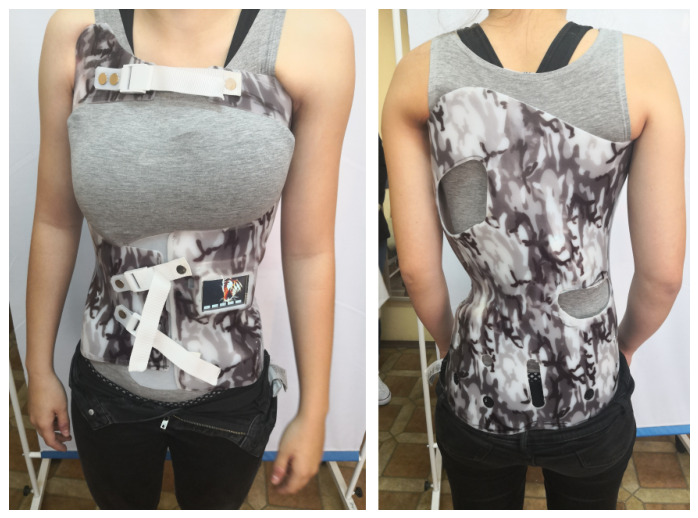
First patient’s brace during testing.

Two types of braces were used during therapy: a temporary brace (Figure 4), worn during the first month, and the target brace (Figure 5) for long-term use. Both were intended not only to stabilize the spine but also to prevent progression of the curvature during the patient’s period of rapid growth. Two braces were used to accommodate both short-term stabilization and long-term corrective treatment. The temporary brace was applied during the first month of therapy to ensure initial stabilization and allow the patient to adapt to brace wearing. The target (long-term) brace was then used for ongoing correction over several months. The decision to use two braces, as well as the timing of their application, was made by the clinician responsible for brace selection and treatment management. For the second brace, a new body scan (plaster cast) was performed to create a custom-fitted orthosis that accurately reflected the patient’s body morphology after initial adaptation and any early changes in spinal curvature. This ensured optimal sensor placement and effective distribution of corrective forces.

The force sensors embedded in the braces enabled detailed analysis of pressure distribution and real-time assessment of correction dynamics and precise adaptation of therapy to treatment progress. Temporary brace sensor placement:

- Sensor 1: left side of the thorax—monitoring thoracic segment stabilization,
- Sensor 2: right paraspinal region—recording pressure at the apex of the curve,
- Sensor 3: right lateral side, adjacent to Sensor 2—analyzing forces in the lateral deformity region,
- Sensor 4: below the left scapula—complementing measurements on the side opposite the curvature.

Target brace sensor placements were as follows: Sensor 1 on the right lateral side, Sensor 2 on the right paraspinal side, Sensor 3 on the left thoracic side, and Sensor 4 below the left scapula.

#### 2.5.2. Case 2: Double-Curve Scoliosis—22° and 18°

**Figure 6:**
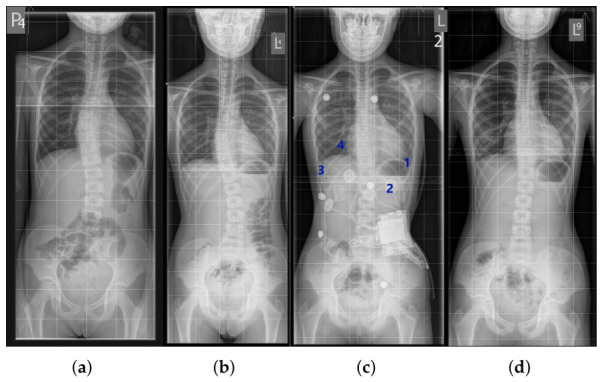
X-ray image of the second patient’s spine: (**a**) At the start of treatment. (**b**) During therapy. (**c**) In the orthopedic brace—full correction visible. (**d**) Without the brace—correction maintained.

An 11-year-old female patient was diagnosed with double-curve scoliosis: a right thoracic curve (Th7-Th12, Cobb angle 22°) and a left lumbar curve (Th12-L3, Cobb angle 18°). In-brace radiograph at the initiation of treatment showed a reduction in the Cobb angle to 9°. Bone maturity, assessed using the Risser scale, was at level 2. Vertebral rotation at the curve apices measured grade 1 for the thoracic curve and grade 2 for the lumbar curve, using the qualitative Cobb-based method [11,12] (Figure 6a).

The first follow-up (Figure 6b) showed maintenance of the thoracic angle (22°) and a slight improvement in the lumbar angle (17°), indicating moderate stabilization. After one year of consistent brace use, full correction of both curves was achieved during therapy (Figure 6c), confirming treatment efficacy and optimal distribution of corrective forces. The brace was equipped with four force sensors:

- Sensor 1: left thoracic area—monitoring anterior pressure,
- Sensor 2: below the left scapula—measuring pressure in the concave region,
- Sensor 3: lower right side—evaluating forces at the thoracic convexity,
- Sensor 4: right paraspinal area—main point for convexity correction.

This sensor placement allowed analysis of forces in various body positions (standing, sitting, lying), and the sensor data served as a basis for real-time optimization of brace settings. Full correction was maintained after the completion of therapy, as documented on X-ray imaging (Figure 6d). The final outcome confirmed lasting spinal stabilization and the effectiveness of IS treatment using a brace with integrated force monitoring.

#### 2.5.3. Case 3: Double-Curve Scoliosis—27° and 33°

**Figure 7:**
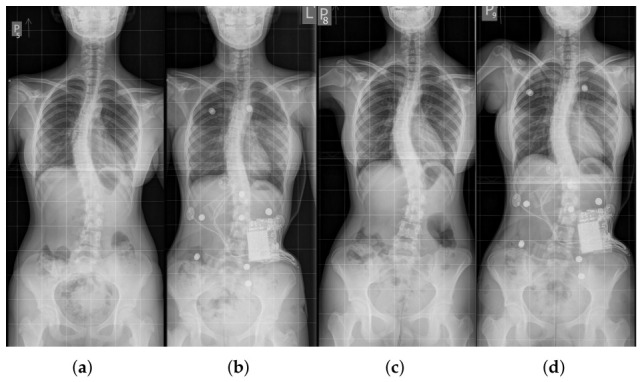
X-ray images of the third patient’s spine: (**a**) At the start of treatment without the brace. (**b**) At the start of treatment in the orthopedic brace. (**c**) During a follow-up visit in the course of therapy without the brace. (**d**) During a follow-up visit in the course of therapy in the orthopedic brace.

A 12-year-old female patient was diagnosed with grade II idiopathic scoliosis of a double-curve nature: right thoracic curvature (27°) and left lumbar curvature (33°), with vertebral rotation at the curve apices of grade 2 for both curves, measured using the qualitative Cobb-based method [11,12] (Figure 7a,b). In-brace radiograph showed partial correction, with Cobb angles of 26° for the thoracic curve and 28° for the lumbar curve. Risser stage 2 indicated an early stage of skeletal maturity, which was significant for potential further progression of the deformity and the effectiveness of orthotic therapy.

Following initiation of brace treatment, moderate correction was observed during follow-up (Figure 7c,d), with both Cobb angles reduced to 26°, suggesting stabilization of the curvatures and potential for further improvement. The brace functioned on a three-point support principle, applying pressure to the convex regions of the curves while offloading the concave areas, allowing controlled correction of spinal geometry.

**Figure 8:**
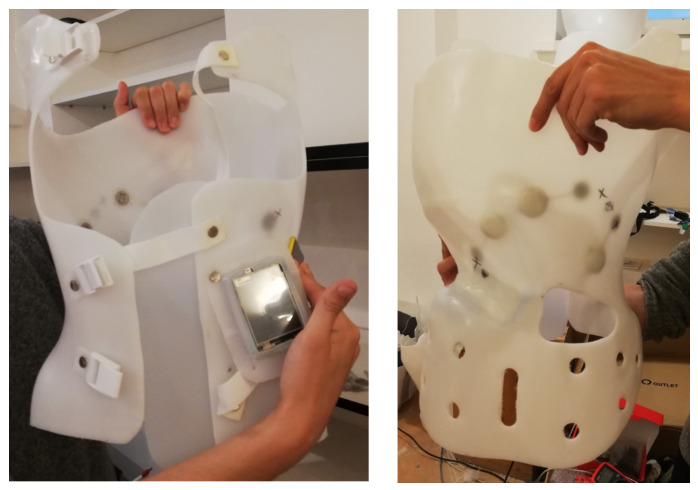
Third patient’s brace with the measurement system installed.

Figure (Figure 8) shows the patient’s custom-fitted brace, equipped with four measurement sensors. Their placement allowed precise monitoring of corrective forces at key points:

- Sensor 1—left thoracic side: monitoring thoracic convexity,
- Sensor 2—below the left scapula: monitoring corrective forces in the lumbar curve,
- Sensor 3—below the right scapula, paraspinal: controlling thoracic rotation and stabilization,
- Sensor 4—lateral, below the right scapula: evaluating uniformity of pressure on the right side.

The collected data enabled assessment of correction effectiveness under real-world conditions and allowed adaptation of brace settings in response to dynamic changes in the patient’s musculoskeletal system.

## 3. Results

### 3.1. Analysis of Corrective Force Distribution—Patient 1

**Figure 9:**
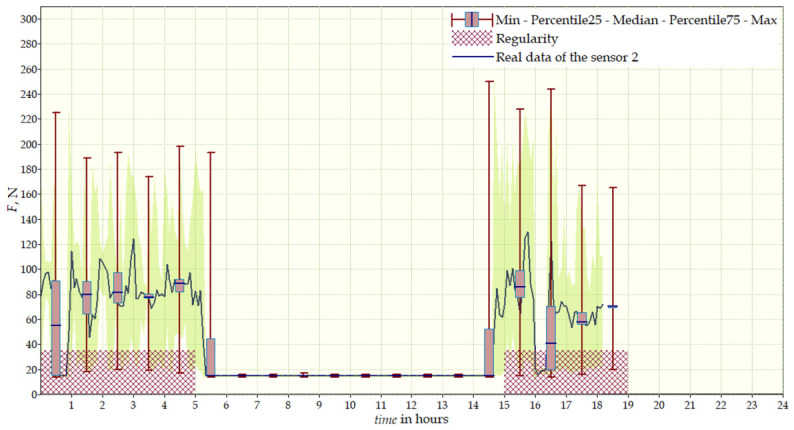
Daily data for Sensor 2 from the first patient’s target brace.

During the initial phase of therapy, the patient wore the temporary brace for 15 days. Sensor data revealed an uneven distribution of forces, with average values as follows: Sensor 1—50 N (max. 240 N), Sensor 2—15 N (max. 60 N), Sensor 3—30 N (max. 160 N), Sensor 4—18 N (max. 30 N), indicating limited correction effectiveness in this phase. After introducing the target brace (worn for 37 days), clear changes in force distribution were recorded: Sensor 1—25 N (max. 240 N), Sensor 2—50 N (max. 280 N), Sensor 3—17 N (max. 80 N), Sensor 4—65 N (max. 160 N). Higher values in Sensors 2 and 4 reflect more effective stabilization of the spine in regions corresponding to the convexity of the curvature. Daily charts (Figure 9) revealed irregular brace use—the average daily wearing time was approximately 8 h, significantly below the recommended 23 h. Possible malfunction of Sensor 4 was observed during the initial phase, requiring system replacement. Figure also includes the regularity parameter, which represents the effective duration of the therapy. The dynamics of corrective forces varied throughout the day due to patient movement, changes in body position, and muscle tension. The target brace, due to better fit, provided more stable forces at key points of the curvature, directly contributing to improved therapeutic effectiveness. Comparison of corrective force values between the temporary and target braces is presented in Table 2.

### 3.2. Analysis of Corrective Force Distribution—Patient 2

**Figure 10:**
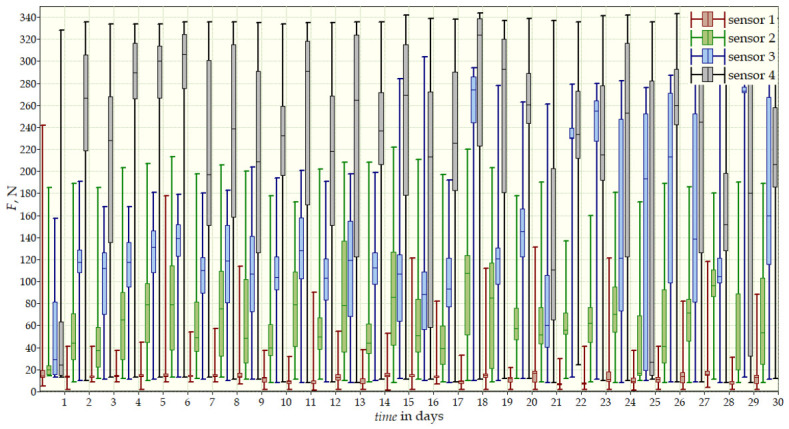
Measurement data from the beginning of the second patient’s therapy for 30 days for all sensors simultaneously.

**Figure 11:**
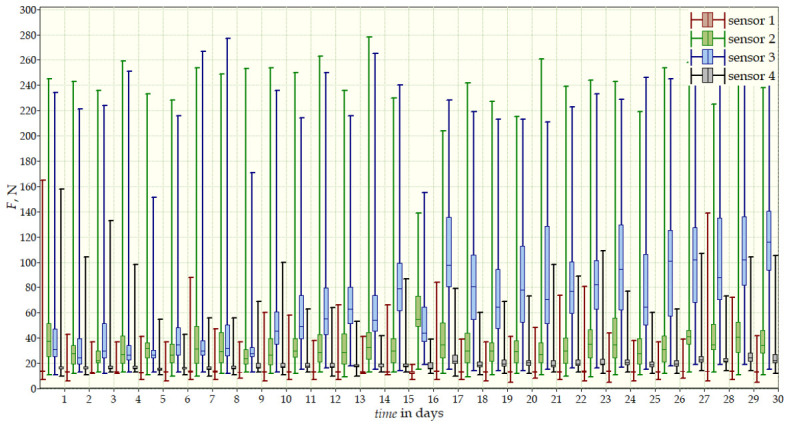
Measurement data from 30 days after one year of therapy for the second patient for all sensors simultaneously.

Measurements were conducted over more than 12 months and included three stages: the beginning of therapy, a 30-day follow-up, and final measurements after one year. The values recorded by the sensors provided a comprehensive view of the dynamics of corrective forces and treatment effectiveness. The results from the start (Figure 10) and after 12 months of therapy (Figure 11) are presented in Table 3. After one year of therapy values decreased significantly. The reduction in forces, particularly at Sensor 4, reflected effective correction of the thoracic curvature. The data also indicated high adherence with brace use, with daily interruptions not exceeding one hour.

### 3.3. Analysis of Corrective Force Distribution—Patient 3

For this patient with double-curve scoliosis, measurements were conducted over two periods: daily and monthly (Table 4). Both datasets revealed substantial fluctuations in forces and uneven distribution, indicating variable brace influence on the spine. Daily measurements showed that Sensor 1 (thorax) recorded a mean force of 300 N and a maximum of 335 N. Sensor 2 (left scapula) measured a mean force of 270 N and a maximum of 315 N. For Sensor 3 (right scapula), the mean value was 120 N, with a maximum of 310 N. Sensor 4 (lower right torso) registered a mean force of 80 N and a maximum of 170 N. Between 04:00 and 18:00, absence of recorded forces suggested that the brace was not worn, limiting therapy effectiveness. The highest forces were recorded in the thoracic region (Sensor 1) and below the left scapula (Sensor 2).

**Figure 12:**
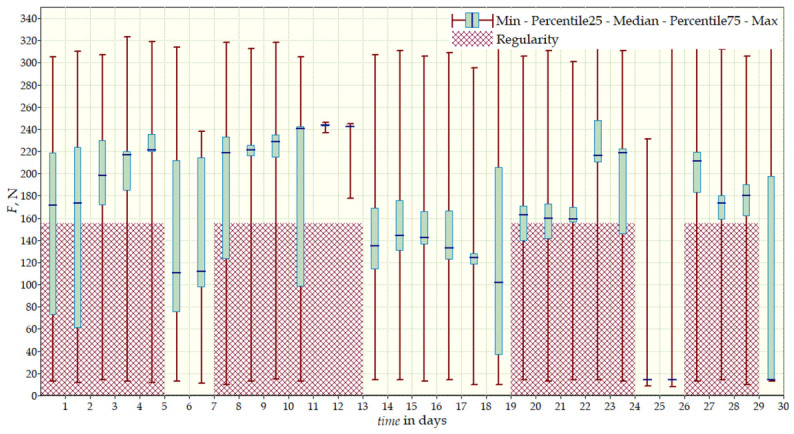
Third patient’s measurement data for Sensor 3 from 30 days.

Monthly measurements (Figure 12) indicated that Sensor 1 recorded a mean force of 260 N and a maximum of 300 N. Sensor 2 measured a mean of 160 N and a maximum of 330 N. Sensor 3 showed a mean force of 170 N and a maximum of 325 N, while Sensor 4 recorded a mean value of 50 N and a maximum of 200 N.

Long-term analysis indicated that Sensor 2 consistently showed higher values, while Sensor 4 still reflected insufficient corrective forces in the lower spine. Variability in forces likely resulted from irregular brace use, physical activity, and the fit of the brace relative to the patient’s anatomical structure.

### 3.4. Adherence Reporting

Adherence data for each patient, calculated over the measurement period, are summarized in Table 5. Patient 1 wore the brace for an average of 8 h per day (35% of the recommended time), resulting in irregular corrective forces. Patient 2 maintained high adherence, wearing it 22 h per day (96%), with consistent force distribution. Patient 3 showed moderate adherence, averaging 14 h per day (61%), with some periods of absent corrective forces. Periods affected by sensor malfunctions were excluded from the analysis.

Patient 1’s low adherence likely limited corrective force application and may have reduced therapy effectiveness, while Patients 2 and 3 achieved higher adherence and more consistent correction. These data indicate that brace effectiveness depends not only on corrective force magnitude but also on adherence to the prescribed wear schedule. Periods of non-adherence corresponded to decreased corrective forces, particularly at sensors monitoring the concave side of the curves, highlighting the clinical relevance of continuous objective monitoring.

## 4. Discussion

Analysis of measurement data from these three clinical cases suggests a potential correlation between the regularity of brace use and the observed force distribution, consistent with the trends reported in larger studies [13,14], which reported that higher daily adherence improves radiological outcomes and overall correction. Patient 1 exhibited low adherence, wearing the brace for only 35% of the prescribed time, which was associated with irregular corrective forces and limited short-term correction. In contrast, Patients 2 and 3 maintained higher adherence (96% and 61%, respectively), allowing more consistent distribution of corrective forces. Variability in forces recorded by the sensors also reflected individual anatomical characteristics and brace design.

The high force values recorded in some sensors, particularly in Patient 3, represent transient peaks occurring during normal daily activity and postural changes rather than constant pressure. The distribution of force across the brace–trunk interface reduces the resulting pressure (kPa) on the skin, ensuring patient comfort and safety. Forces were continuously evaluated by the treating clinician and orthotist, who adjusted the brace to maintain effective correction while avoiding discomfort or skin complications. No adverse events, such as skin irritation, pain, or tissue damage, were observed during the monitoring period.

These observations underscore that the recorded forces reflect real-world usage rather than maximal tolerable limits. Future studies could benefit from integrating contact-area measurements or pressure mapping to quantify local pressures more accurately and correlate them with biomechanical safety thresholds.

Table 6 summarizes the corrective forces (mean and maximum values) recorded by the sensors during 24-h monitoring in the three clinical cases. Force values differed depending on sensor location, reflecting individual anatomical features, brace fit, and adherence to therapeutic recommendations. The highest forces were recorded in the thoracic region and beneath the apex of the thoracic curve, while the lowest forces were observed in the posterior-lateral torso (Sensor 4), suggesting that, within this specific pilot group, that area received lower mechanical loading, which may indicate a need for further optimization of brace fit in similar cases.

At the same time, sensor data revealed a number of practical issues: insufficient brace fit in specific areas, technical challenges in achieving even force distribution, and irregular brace use. In particular, low force values recorded by Sensor 4 in some cases indicated the need for brace design adjustments or refitting.

An important aspect discussed in this study is the irregularity of brace use, which directly influenced temporal variability in corrective forces. Days during which the sensors recorded no values confirmed non-adherence with therapeutic recommendations, potentially limiting treatment outcomes. Adherence was quantified based on sensor-recorded forces exceeding a threshold of 5 N. Periods below this threshold for more than 30 min were considered non-wear. This allowed calculation of daily adherence percentages, providing an objective measure of brace-wearing regularity alongside the observed force patterns, in line with evidence that adherence and in-brace correction rate influence Cobb angle progression [15]. Thus, measurement analysis not only allows assessment of the biomechanical aspects of correction but also serves as a tool for monitoring adherence to clinical guidelines. Brace-wearing regularity was inferred indirectly from force recordings. The system did not include an independent temperature-based adherence monitor, nor was brace-wearing time systematically compared with patient diaries. Therefore, adherence assessment should be interpreted with caution.

Currently, there is no established consensus regarding optimal magnitude or localization of corrective forces in brace treatment of IS. The relationship between applied force and biological response of growing vertebrae remains insufficiently defined. Therefore, interpretation of recorded force values in this study should be considered exploratory rather than normative.

The development of modern orthopedics is increasingly moving toward personalized therapy, in which the analysis of biometric data plays a key role in supporting clinical decision-making and enhancing therapeutic effectiveness. Contemporary advances in AI are widely applied in medical imaging diagnostics, surgical planning, and rehabilitation monitoring, enabling dynamic adjustments to treatment protocols in response to the patient’s actual needs, thereby improving therapeutic outcome [16,17,18]. This approach is particularly important in the treatment of conditions requiring long-term biomechanical intervention, such as IS, where the dynamic adjustment of corrective forces is critical to the effectiveness of treatment.

The complexity of mathematically modeling idiopathic scoliosis arises from high morphological variability and dynamic progression. Addressing these challenges requires the use of advanced analytical and algorithmic tools for effective classification and identification of diagnostic and therapeutic rules [19,20]. Measurement accuracy is also critical, as Force Sensitive Resistor (FSR) sensors exhibit nonlinear response, hysteresis, and temperature drift, necessitating careful calibration and compensation [21]. Integration of sensor systems with machine learning and data fusion techniques has enabled prediction of biomechanical forces and personalized mapping of mechanical loads, converting raw brace data into actionable therapeutic metrics [22,23].

Previous studies have demonstrated the potential of wearable sensors in biomechanical monitoring. Zhao et al. [24] developed a flexible sensor matrix placed on a thin film and positioned on the shoe insole, which combined with a measurement and transmission system enables the measurement and visualization of pressure distribution across different foot zones during walking. Similarly, the system presented by Wang et al. [25], integrating inertial measurement units (IMU) with pressure sensors, allows the simultaneous measurement of both kinematic and kinetic parameters during human gait. In a pilot study monitoring force and temperature inside an orthopedic brace during IS treatment, Zou et al. [26] used an integrated data logger equipped with a force sensor (FS1500) and a temperature sensor, which enabled strict control of the therapeutic regimen and enhanced its effectiveness. These developments support the clinical relevance of real-time force monitoring in smart braces.

Sensor systems applied in orthopedic rehabilitation have already demonstrated their potential in clinical practice. Patel et al. [27] emphasized the role of wearable devices in monitoring patients at home and in telemedicine, highlighting their ability to collect real-world data and support clinical decision-making. Similarly, Yang et al. [28] pointed out the importance of comfort, ease of use, and patient acceptance in the design of wearable systems, which is particularly critical for therapies that require prolonged brace use.

Compared to temperature-based adherence monitoring systems [4], the presented solution additionally provides information on the magnitude and localization of corrective forces. Unlike systems limited to wear-time assessment, our approach enables biomechanical characterization of brace–trunk interaction. A limitation of the present system is that sensors were positioned only in selected corrective areas. Although these points correspond to the main pressure zones of a Chêneau-type brace, localized measurement may not fully reflect the global three-dimensional force distribution. Consequently, some corrective interactions brace and trunk may not have been captured.

Recent clinical studies further support the role of adherence in determining brace effectiveness. Pjanic et al. [13] used thermal sensors to monitor brace wear and found a strong correlation between adherence and radiological outcomes. Donzelli et al. [14] demonstrated that consistent daily brace use improves correction results in a case-control study. Sakashita et al. [15] highlighted that skeletal maturity, compliance, and in-brace correction rate are key predictors of Cobb angle progression. These findings are consistent with the patterns observed in our pilot data, suggesting that real-world adherence plays a key role in the stability of corrective forces, although the small sample size precludes definitive clinical generalizations.

The results support the rationale for using real-time measurement systems as a tool to assist therapeutic decision-making. The ability to monitor force distribution and detect irregularities in brace use enables real-time adjustment of orthosis settings, which is particularly critical during periods of rapid skeletal growth in patients. Comparison of short-term and long-term data shows that corrective forces are unstable and highly variable. This underscores the need for more advanced mechanisms to adapt the brace to dynamic usage conditions, as well as strict real-time therapy monitoring, which could enhance treatment effectiveness.

The presented clinical cases confirm the growing potential of smart braces equipped with biomechanical data acquisition and analysis systems, aligning with the broader trend of wearable technology development in medicine. Integration of flexible sensors allows not only precise monitoring of movement and posture but also greater comfort due to anatomical adaptation to the patient’s body [29]. Combined with AI algorithms and telemedicine platforms, such solutions can support therapy personalization, improve adherence, and enable early detection of curvature progression [29,30]. Therefore, future research should focus on clinical validation, optimization of materials and algorithms, and the development of standards that allow scalable implementation of smart braces in routine orthopedic practice.

### Limitations

The present study has several limitations. First, the number of participants was limited to three patients, reflecting the pilot and feasibility nature of the study. While the number of participants is small (*n* = 3), we would like to emphasize that the study provides a massive dataset of over 10,000 h of continuous monitoring. This allowed us to capture a range of patient behaviors from high (96%) to lower adherence (35%). Such diversity in real-world data is critical for validating the robustness of the sensor system and developing machine learning models that must handle various usage patterns, which was the primary technical goal of this study. Second, sensor placement, although guided by biomechanical analysis and the attending physician’s experience, may vary slightly due to individual anatomy and brace fitting. Third, potential measurement errors could arise from patient movement, incorrect brace fastening, or sensor calibration drift. Finally, force data were recorded only at selected key locations of the brace, which may not fully capture the global three-dimensional distribution of corrective forces.

The primary objective of this study was to evaluate the technical performance of the integrated force-sensor system, rather than to assess long-term clinical effectiveness. Patients were monitored over periods ranging from several months to over a year to analyze corrective force distribution and adherence. Long-term outcomes, such as final Cobb angles or vertebral rotation after treatment, were not collected, reflecting the pilot technical nature of the study.

## 5. Conclusions

The use of a smart orthosis enables dynamic analysis of treatment progression and effectiveness, as well as improved adaptation of the device to the patient’s needs. The results suggest the necessity for further development of predictive models and personalized therapy.

In the analyzed cases, periods of zero recorded forces were observed, which illustrate how irregular brace use could potentially limit the biomechanical impact of the therapy. Uneven distribution of corrective forces, particularly low values near Sensor 4, points to the need for further customization of the orthosis design according to the patient’s individual anatomical characteristics. Such uneven force distribution may result from both design limitations and individual anatomical differences.

Recorded force fluctuations were influenced by multiple factors, including changes in body position, physical activity levels, and brace fastening technique, emphasizing the importance of patient education regarding proper orthosis use. Treatment effectiveness largely depends on the regularity and accuracy of brace wear.

Real-time pressure monitoring via force sensors provides a valuable tool both for assessing the biomechanical effectiveness of treatment and for monitoring adherence to therapeutic recommendations. Long-term monitoring allows for detection of irregularities and optimization of brace function.

While this technical pilot was not designed to statistically evaluate clinical outcomes, the observed force stability in the high-adherence patient (Patient 2) provides a promising baseline for future large-scale trials. The monitoring system did not directly influence therapeutic results, it provides significant added value by enabling future determination of reference force values suitable for different curve types and skeletal ages to achieve optimal outcomes. The system also enables the creation of a database to support future development of predictive models and treatment planning.

Future work should focus on optimizing sensor placement, automated data analysis, and real-time personalization of brace settings to increase treatment effectiveness and improve patient comfort. The findings underscore the need for continued development of intelligent orthotic systems, particularly toward automatic real-time adjustment of corrective forces in response to dynamic anatomical and biomechanical conditions of the patient. 

## Figures and Tables

**Table 1:** Comparison between the previous prototype [10] and the current monitoring system.

Feature	Previous System [10]	Current Study
Monitoring Duration	Short-term technical tests	Long-term (even >1 year), multiple patients
Data Volume	Limited samples	>10,000 h of continuous data
Power Management	Required frequent recharging	Battery life > 1 month, maintenance-free; improved energy efficiency
System Architecture	Single integrated unit	Two separate modules (brace-embedded node + gateway); sensor redesigned
Data Transmission	Intermittent GSM connection	Continuous wireless via gateway
Clinical Validation	Single case/Technical proof	3 patients (diverse adherence)
Application Goal	Basic force recording	High-quality longitudinal dataset for ML/AI, supporting predictive modeling

**Table 2:** Comparison of corrective force values (N)—temporary vs. target brace.

Sensor Number	Temporary Brace Mean/Max, N	Target Brace Mean/Max, N	Comment
Sensor 1	50/240	25/240	Shift of forces from the anterior torso to corrective areas
Sensor 2	15/60	50/280	Significant increase in pressure at the curve apex
Sensor 3	30/160	17/80	Reduced lateral forces, correction focused closer to the spine
Sensor 4	18/30	65/160	Stronger stabilization on the concave side

**Table 3:** Comparison of corrective forces in Patient 2—beginning vs. after 12 months of therapy.

Sensor Number	Start of Therapy Mean/Max, N	After 12 Months Mean/Max, N	Change/Comment
Sensor 1	18/240	18/160	No change in mean, decrease in maximum
Sensor 2	60/220	35/260	Decrease in mean, increase in maximum force
Sensor 3	160/300	70/270	Significant reduction in mean values
Sensor 4	260/340	20/160	Strong reduction in pressure, effective curve correction

**Table 4:** Comparison of corrective forces in Patient 3—daily vs. monthly data.

Sensor Number	Daily Data Mean/Max, N	Monthly Data Mean/Max, N	Comment
Sensor 1	300/335	260/300	High and stable pressure values
Sensor 2	270/315	160/330	Intensive corrective action on the lumbar curve
Sensor 3	120/310	170/325	Variability indicating irregular loading
Sensor 4	80/170	50/200	Lowest values—needed for better calibration

**Table 5:** Objective adherence assessment based on sensor data.

Patient	Average Daily Wear, h	Adherence, %
1	8	35
2	22	96
3	14	61

**Table 6:** Corrective force summary (daily range).

Sensor Number	Location	Patient 1, N	Patient 2, N	Patient 3, N
Sensor 1	Anterior thoracic wall	50–240	40–160	300–335
Sensor 2	Below the left scapula	15–60	60–260	270–315
Sensor 3	Right scapular area	30–160	70–270	120–310
Sensor 4	Below the right scapula (lateral)	18–30	20–160	80–170

## Data Availability

The datasets analyzed during the current study are available from the corresponding author on request.

## Abbreviations

The following abbreviations are used in this manuscript:

AI	Artificial Intelligence
IS	Idiopathic Scoliosis
FSR	Force Sensitive Resistor
IMU	Inertial Measurement Units
SOSORT	Society on Scoliosis Orthopaedic and Rehabilitation Treatment
TLSO	Thoracolumbosacral Orthosis
